# miR-16 promotes the apoptosis of human cancer cells by targeting FEAT

**DOI:** 10.1186/s12885-015-1458-8

**Published:** 2015-06-02

**Authors:** Hongwei Liang, Zheng Fu, Xueyuan Jiang, Nan Wang, Feng Wang, Xueliang Wang, Suyang Zhang, Yanbo Wang, Xin Yan, Wen-xian Guan, Chen-Yu Zhang, Ke Zen, Yujing Zhang, Xi Chen, Guangxin Zhou

**Affiliations:** 1 Department of Orthopedics, Jinling Hospital, School of Medicine, Nanjing University, 305 East Zhongshan Road, Nanjing, Jiangsu 210002 China; 2 Jiangsu Engineering Research Center for microRNA Biology and Biotechnology, State Key Laboratory of Pharmaceutical Biotechnology, School of Life Sciences, Nanjing University, 22 Hankou Road, Nanjing, Jiangsu 210093 China; 3 The Comprehensive Cancer Center of Drum Tower Hospital Affiliated to Medical School of Nanjing University & Clinical Cancer Institute of Nanjing University, Nanjing, Jiangsu 210008 China; 4 Department of Pathology, Keck School of Medicine, University of Southern California, Los Angeles, CA 90033 USA; 5 Department of General Surgery of Drum Tower Hospital Affiliated to Medical School of Nanjing University, Nanjing, Jiangsu 210008 China

**Keywords:** miR-16, FEAT, Apoptosis

## Abstract

**Background:**

Although human cancers have heterogeneous combinations of altered oncogenes, some crucial genes are universally dysregulated in most cancers. One such gene, *FEAT* (faint expression in normal tissues, aberrant overexpression in tumors), is uniformly overexpressed in a variety of human cancers and plays an important role in tumorigenesis by suppressing apoptosis. However, the precise molecular mechanism through which FEAT is upregulated during tumorigenesis remains largely unknown.

**Methods:**

In this study, we used bioinformatic analyses to search for miRNAs that potentially target FEAT. We examined the expression of FEAT protein level by western blotting and miR-16 level by qRT-PCR assay. Cancer cell lines (A549, MCF-7 and Huh-7) with miR-16 upregulation and FEAT silencing were established and the effects on apoptosis of cancer cells *in vitro* were assessed. Luciferase reporter assay was also performed to investigate the interaction between miR-16 and FEAT.

**Results:**

We identified a specific target site for miR-16 in the 3′-untranslated region (3′-UTR) of FEAT. Consistent with the bioinformatic analyses, we identified an inverse correlation between the miR-16 and FEAT protein levels in lung cancer, breast cancer, and hepatocellular cancer tissues. We then experimentally validated miR-16 as a direct regulator of FEAT using cell transfection and luciferase assays. Finally, we demonstrated that the repression of FEAT by miR-16 promoted the apoptosis of cancer cells.

**Conclusions:**

Our findings provide the first clues regarding the role of miR-16 as a tumor suppressor in cancer cells through the inhibition of FEAT translation.

**Electronic supplementary material:**

The online version of this article (doi:10.1186/s12885-015-1458-8) contains supplementary material, which is available to authorized users.

## Background

Although our understanding of the molecular mechanisms of carcinogenesis has greatly improved, this knowledge has not led to the identification and development of effective tools for cancer screening and prevention. In theory, one of the most feasible and promising approaches for cancer screening and prevention is targeting a common oncogene that occurs in most tumors. However, the marked heterogeneity and complexity of human tumors make it difficult to identify commonalities among cancers [1, 2]. Oncogenes that contribute to the development of human cancers are highly variable among different types of cancer and among individual tumors of the same type [1, 2]. Thus, it is still poorly understood whether there are crucial oncogenes that are commonly altered in diverse cancers. Recently, Takahashi et al. investigated a previously unrecognized protein, FEAT (faint expression in normal tissues, aberrant overexpression in tumors), and identified it as a novel, prominent promoter of tumorigenesis [3]. FEAT protein is encoded by *METTL13* gene (methyltransferase like 13), and it is aberrantly overexpressed in most human cancers but weakly expressed in normal tissues [3]. Remarkably, transgenic mice that ectopically expressed FEAT spontaneously developed tumors, indicating that the FEAT protein potently drives tumorigenesis *in vivo* [3]. Gene expression profiling has suggested that FEAT drives receptor tyrosine kinase and hedgehog signaling pathways [3]. However, despite these recent advances in our understanding of the important roles of FEAT in cancer progression, the precise molecular mechanism through which FEAT is upregulated during tumorigenesis remains largely unknown. Further studies are needed to fully elucidate the regulation of FEAT expression in normal and neoplastic tissues.

microRNAs (miRNAs) are a class of endogenously expressed, small non-coding RNAs that play an important role in the regulation of gene expression at the post-transcriptional level [4–6]. Some of these miRNAs have attracted special attention for their involvement in the initiation, progression, and metastasis of human cancers [7, 8]. One particularly well-studied example is the ubiquitously expressed and highly conserved miR-16, one of the first miRNAs that was known to be linked to human malignancies [9]. Evidence indicates that miR-16 can modulate the cell cycle, inhibit cell proliferation, promote cell apoptosis, and suppress tumorigenicity both *in vitro* and *in vivo* [10]. These effects can be explained by several targets of miR-16: the anti-apoptotic gene B-cell lymphoma 2 (*Bcl-2*) [11]; numerous genes involved in the G1-S transition such as *CCND1* (cyclin D1), *CCND3* (cyclin D3), *CCNE1* (cyclin E1), and *CDK6* (cyclin-dependent kinase 6) [12–14]; and genes involved in the Wnt signaling pathway, such as *WNT3A* (wingless-type MMTV integration site family, member 3A) [14]. Consistently, miR-16 is frequently deleted and/or downregulated in many types of cancer, including chronic lymphocytic leukemia [9, 15], prostate cancer [14], and lung cancer [16]. Thus, miR-16 is generally thought to be a key tumor-suppressive miRNA that can target numerous oncogenes in various human cancers.

Although the dysregulation of miR-16 and FEAT plays an important role in carcinogenesis, no correlation between FEAT and miR-16 in cancers has been reported. In this study, we hypothesized that FEAT is a target of miR-16. After measuring the expression levels of miR-16 and FEAT in different types of human cancer tissues and paired noncancerous tissues, we detected an inverse correlation between miR-16 and FEAT in human cancers. Furthermore, in this study, we experimentally investigated the direct regulation of FEAT by miR-16 and the biological role of miR-16 targeting FEAT in human cancer cells.

## Methods

### Cells and human tissues

The human lung cancer cell lines A549, human breast cancer cell lines MCF-7, and human liver cancer cell lines Huh-7 were purchased from the Shanghai Institute of Cell Biology, Chinese Academy of Sciences (Shanghai, China). A549, MCF-7, and Huh-7 cells were cultured in DMEM supplemented with 10 % fetal bovine serum (GIBCO, CA, USA). All cells were incubated in a 5 % CO_2_ at 37 °C in a water-saturated atmosphere. The tumors and paired normal adjacent tissues were derived from patients undergoing a surgical procedure at the Affiliated Gulou Hospital of Nanjing University (Nanjing, China). All of the patients or their guardians provided written consent, and the Ethics Committee from Nanjing University approved all aspects of this study. Tissue fragments were immediately frozen in liquid nitrogen at the time of surgery and stored at −80 °C. The clinical features of the patients are listed in Table 1.

**Table 1:** Clinical Features of cancer patients

Cancer type	Tumor subtype	Pathological stage
Lung cancer #1	Adenocarcinoma	IIIA
Lung cancer #2	Adenocarcinoma	IIB
Lung cancer #3	Squamous cell carcinoma	IIIA
Breast cancer #1	Invasive Ductal Carcinoma	III
Breast cancer #2	Invasive Ductal Carcinoma	III
Breast cancer #3	Invasive Ductal Carcinoma	II
Hepatocellular cancer #1	Hepatocellular carcinoma	II
Hepatocellular cancer #2	Hepatocellular carcinoma	II
Hepatocellular cancer #3	Hepatocellular carcinoma	III

### RNA isolation and quantitative RT-PCR

Total RNA was extracted from the cultured cells and human tissues using TRIzol Reagent (Invitrogen, Carlsbad, CA) according to the manufacturer’s instructions. Assays to quantify miRNAs were performed using Taqman miRNA probes (Applied Biosystems, Foster City, CA) according to the manufacturer’s instructions. Briefly, 1 μg of total RNA was reverse-transcribed to cDNA using AMV reverse transcriptase (TaKaRa, Dalian, China) and a stem-loop RT primer (Applied Biosystems). The reaction conditions were as follows: 16 °C for 30 min, 42 °C for 30 min, and 85 °C for 5 min. Real-time PCR was performed using a TaqMan PCR kit on an Applied Biosystems 7300 Sequence Detection System (Applied Biosystems). The reactions were incubated in a 96-well optical plate at 95 °C for 10 min, followed by 40 cycles of 95 °C for 15 s and 60 °C for 1 min. All of the reactions were run in triplicate. After the reaction, the cycle threshold (C_T_) data were determined using fixed threshold settings, and the mean C_T_ of the triplicate PCRs was determined. A comparative C_T_ method was used to compare each condition with the controls. The relative levels of the miRNAs in the cells and tissues were normalized to U6. The amount of miRNA relative to the internal control U6 was calculated using the 2^-△△CT^ equation, in which △△C_T_ = (C_T miRNA_ - C_T U6_)_target_—(C_T miRNA_—C_T U6_)_control_. To quantify the FEAT mRNA, 1 μg of total RNA was reverse-transcribed to cDNA using oligo(dT) and Thermoscript (TaKaRa) in the reaction, which was performed under the following conditions: 42 °C for 60 min and 70 °C for 10 min. Next, real-time PCR was performed using the RT product, SYBER Green Dye (Invitrogen) and specific primers for FEAT and GAPDH. The sequences of the primers were as follows: FEAT (sense): 5′—CTTCACCGAGGTCAGCAGTA-3′; FEAT (antisense): 5′—CTCCATGACTCTAGCCGACA-3′; GAPDH (sense): 5′-GATATTGTTGCCATCAATGAC-3′; and GAPDH (antisense): 5′-TTGATTTTGGAGGGATCTCG-3′. The reactions were incubated at 95 °C for 5 min, followed by 40 cycles of 95 °C for 30 s, 60 °C for 30 s, and 72 °C for 30 s. After the reactions were complete, the C_T_ values were determined by setting a fixed threshold. The relative amount of FEAT mRNA was normalized to GAPDH.

### Overexpression and knockdown of miR-16

Synthetic pre-mir-16, anti-mir-16, and scrambled negative control RNAs were purchased from Ambion (Austin, TX, USA). All cells were seeded in 6-well plates or 60-mm dishes, and the cells were transfected with Lipofectamine 2000 (Invitrogen) on the following day, when the cells were approximately 70 % confluent. In each well, equal amounts of pre-mir-16, anti-mir-16, or scrambled negative control RNA were used. The cells were harvested 24 h after transfection for quantitative RT-PCR and Western blotting.

### Luciferase reporter assay

To test the direct binding of miR-16 to the target gene FEAT, a luciferase reporter assay was performed as previously described [17]. The entire 3′-untranslated region (3′-UTR) of human FEAT was amplified using PCR with human genomic DNA as a template. The PCR products were inserted into the p-MIR-reporter plasmid (Ambion), and the insertion was confirmed to be correct via sequencing. To test the binding specificity, the sequences that interacted with the miR-16 seed sequence were mutated (from UGCUGCU to ACGACGA), and the mutant FEAT 3′-UTR was inserted into an equivalent luciferase reporter. For luciferase reporter assays, A549, MCF-7, and Huh-7 cells were cultured in 24-well plates, and each well was transfected with 1 μg of firefly luciferase reporter plasmid, 1 μg of a β-galactosidase (β-gal) expression plasmid (Ambion), and equal amounts (100 pmol) of pre-mir-16, anti-mir-16, or the scrambled negative control RNA using Lipofectamine 2000 (Invitrogen). The β-gal plasmid was used as a transfection control. Twenty-four hours post-transfection, the cells were assayed using a luciferase assay kit (Promega, Madison, WI, USA).

### Plasmid construction and siRNA interference assay

An siRNA sequence targeting the human FEAT cDNA was purchased from Santa Cruz (sc-88139, Santa Cruz Biotechnology, CA, USA). A scrambled siRNA was included as a negative control. A mammalian expression plasmid encoding the human FEAT open reading frame (pReceiver-M02-FEAT) was purchased from GeneCopoeia (Germantown, MD, USA). An empty plasmid served as a negative control. The FEAT expression plasmid and FEAT siRNA were transfected into A549 cells using Lipofectamine 2000 (Invitrogen) according to the manufacturer’s instructions. Both total RNA and protein were isolated 24 h post-transfection. The FEAT mRNA and protein expression levels were assessed by quantitative RT-PCR and Western blotting.

### Protein extraction and western blotting

All cells were rinsed with PBS (pH 7.4) and lysed in RIPA Lysis buffer (Beyotime, China) supplemented with a Protease and Phosphatase Inhibitor Cocktail (Thermo Scientific 78440) on ice for 30 min. The tissue samples were frozen solid with liquid nitrogen, ground into a powder and lysed in RIPA Lysis buffer containing the Protease and Phosphatase Inhibitor Cocktail on ice for 30 min. When necessary, sonication was used to facilitate lysis. Cell lysates or tissue homogenates were centrifuged for 10 min (12000 g, 4 °C). The supernatant was collected, and the protein concentration was calculated using a Pierce BCA protein assay kit (Thermo Scientific, Rockford, IL, USA). The protein levels were analyzed using Western blots with the corresponding antibodies. The protein levels were normalized by probing the same blots with a GAPDH antibody. The antibodies were purchased from the following sources: anti-FEAT (X-20) (sc-101995, Santa Cruz Biotechnology, CA, USA) and anti-GAPDH (sc-365062, Santa Cruz Biotechnology, CA, USA). Protein bands were analyzed using the ImageJ software.

### Apoptosis assays

The apoptosis of A549 cells was tested using an Annexin V-FITC/propidium iodide (PI) staining assay. A549 cells were cultured in 12-well plates and transfected with pre-mir-16, anti-mir-16, FEAT siRNA, or the FEAT overexpression plasmid to induce apoptosis. Pre-mir-control, anti-mir-control, control siRNA, and control plasmid served as negative controls. Cells were cultured overnight with both serum-containing complete medium and serum-depleted medium; and the attached and floating cells were then harvested. Flow cytometry analysis of apoptotic cells was performed using an Annexin V-FITC/PI staining kit (BD Biosciences, CA, USA). After washes with cold PBS, the cells were resuspended in binding buffer (100 mM HEPES, pH 7.4, 100 mM NaCl, and 25 mM CaCl_2_), followed by staining with Annexin V-FITC/PI at room temperature in darkness for 15 min. Apoptotic cells were then evaluated by gating PI and Annexin V-positive cells on a fluorescence-activated cell-sorting (FACS) flow cytometer (BD Biosciences, San Jose, CA). All experiments were performed in triplicate.

### Functional annotation

Based on miRTarBase, there are now more than 100 experimental validated target genes of miR-16 [18]. the Gene Ontology (GO) classification was performed to gain insights into the biological functions of miR-16 target genes, and the Kyoto Encyclopedia of Genes and Genomes (KEGG) pathway enrichment analysis was performed to detect the potential pathway of miRNA target genes. KEGG pathway database is a recognized and comprehensive database including all kinds of biochemistry pathways [19]. The online based software GENECODIS was utilized in those functional annotation [20].

### Statistical analysis

All of the Western blotting images are representative of at least three independent experiments. Quantitative RT-PCR, the luciferase reporter assay, and the cell viability and apoptosis assays were performed in triplicate, and each experiment was repeated several times. The data shown are the mean ± SE of at least three independent experiments. The differences were considered statistically significant at p < 0.05 using Student’s *t*-test.

## Results

### Upregulation of FEAT protein in human cancer tissues

**Fig. 1:**
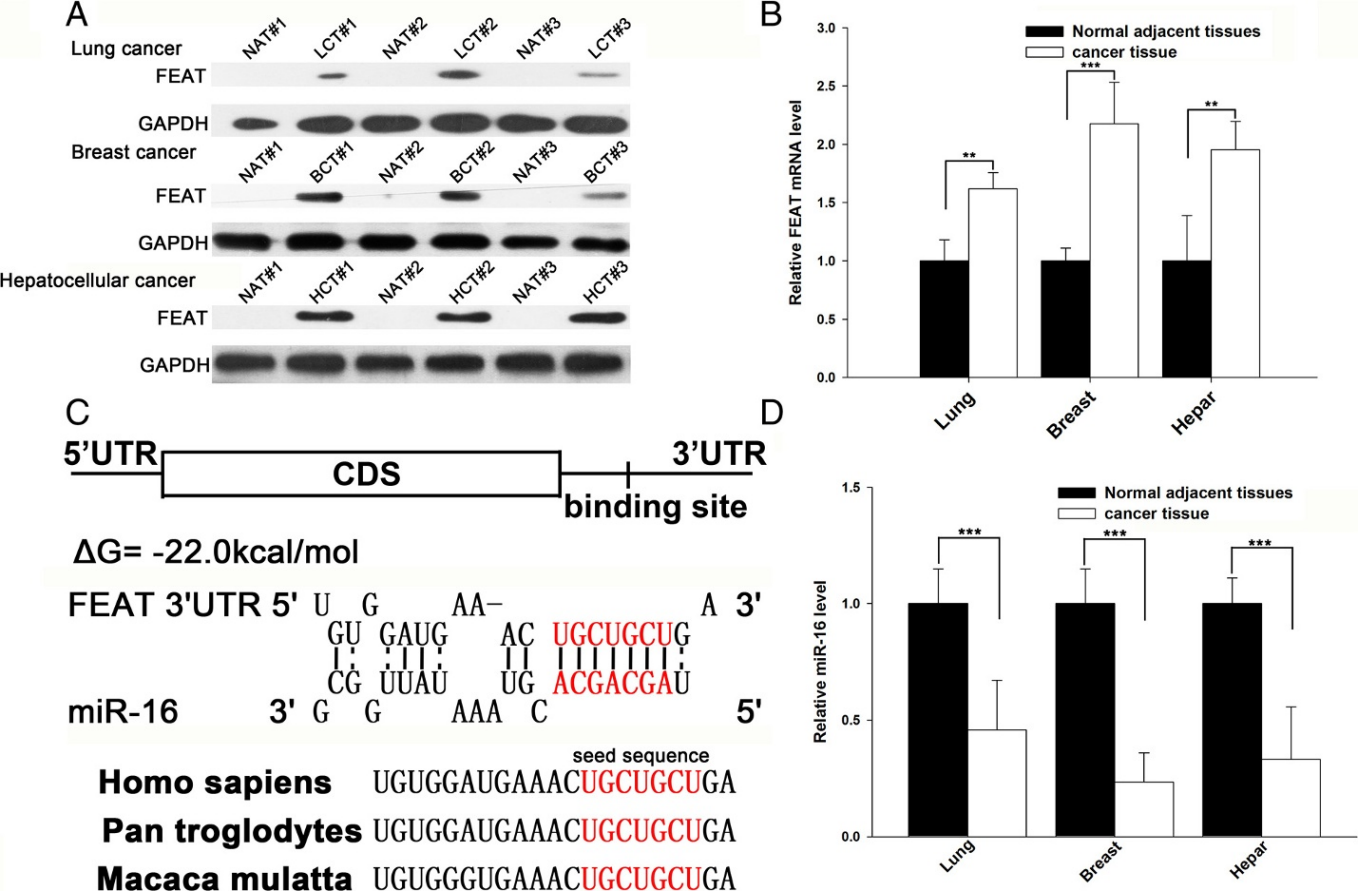
Expression levels of the FEAT protein, FEAT mRNA, and miR-16 in cancer tissues. **a** Western blotting analysis of the expression levels of the FEAT protein in three pairs of lung (LCT), breast (BCT), and hepatocellular cancer tissues (HCT) and in normal adjacent tissues (NAT). **b** Quantitative RT-PCR analysis of the relative expression levels of FEAT mRNA in three pairs of lung, breast, and hepatocellular cancer tissues and in normal adjacent tissues. **c** Schematic description of the hypothetical duplexes formed by the interactions between the binding site in the FEAT 3′-UTR (top) and miR-16 (bottom). The predicted free energy value of the hybrid is indicated. The seed recognition site is denoted, and all nucleotides in this region are highly conserved across species. (D) Quantitative RT-PCR analysis of the expression levels of miR-16 (in the form of the miRNA/U6 ratio) in three pairs of lung, breast, and hepatocellular cancer tissues and in normal adjacent tissues. **, *P* < 0.01; ***, *P* < 0.001

We first determined the expression patterns of FEAT in lung cancer, breast cancer, and hepatocellular cancer tissues. After measuring the protein levels of FEAT in these cancer tissues and the corresponding noncancerous tissues, we found that FEAT protein is dramatically overexpressed in cancer tissues but totally absent in normal tissues (Fig. 1a). However, FEAT mRNA was readily detected in noncancerous tissues, and its levels were slightly upregulated in cancer tissues (Fig. 1b). This disparity between protein and mRNA in FEAT expression in cancers strongly suggests that a post-transcriptional mechanism is involved in FEAT regulation.

### Identification of conserved miR-16 target sites within the 3′-UTR of FEAT

One important mode of post-transcriptional regulation is the repression of mRNA transcripts by miRNAs. miRNAs are therefore likely to play a biologically relevant role in regulating FEAT expression in cancer. Three computational algorithms, including TargetScan [21], miRanda [22], and PicTar [23], were used in combination to identify potential miRNAs that can target FEAT. Using these approaches, miR-16 was identified as a candidate regulatory miRNA of FEAT. The predicted interaction between miR-16 and the target sites in the FEAT 3′-UTR are illustrated in Fig. 1c. One potential miR-16 target site was found in the 3′-UTR of the FEAT mRNA sequence. The minimum free energy value of this hybrid is −22.0 kcal/mol, which is well within the range of genuine miRNA-target pairs. Moreover, perfect base pairing occurs between the seed region (the core sequence that encompasses the first 2–8 bases of the mature miRNA) and the cognate targets. Furthermore, the miR-16 binding sequence in the FEAT 3′-UTR is highly conserved across species.

### Detection of an inverse correlation between the miR-16 and FEAT levels in cancer tissues

miRNAs are generally thought to have expression patterns that are the opposite of those of their targets [4–6]. We next investigated whether miR-16 was inversely correlated with FEAT in cancer tissues. After determining the levels of miR-16 in the same three pairs of lung cancer, breast cancer, and hepatocellular cancer tissues and the corresponding noncancerous tissues, we showed that the miR-16 levels were consistently downregulated in cancer tissues (Fig. 1d). Combining the computational prediction with the detection of inverse correlation between miR-16 and FEAT *in vivo*, it is quite likely that miR-16 is involved in the post-transcriptional regulation of FEAT.

### Validation of FEAT as a direct target of miR-16

**Fig. 2:**
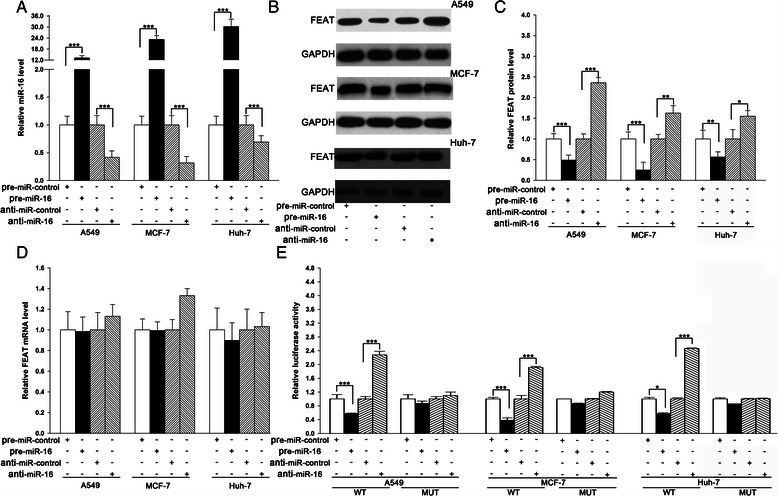
Direct regulation of FEAT expression by miR-16 at the posttranscriptional level. **a** Quantitative RT-PCR analysis of the miR-16 levels in A549, MCF-7, and Huh-7 cells transfected with pre-mir-control, pre-mir-16, anti-mir-control, and anti-mir-16. **b** and **c** Western blot analysis of the FEAT protein levels in A549, MCF-7, and Huh-7 cells transfected with pre-mir-control, pre-mir-16, anti-mir-control, and anti-mir-16. B: representative image; C: quantitative analysis. **d** Quantitative RT-PCR analysis of FEAT mRNA levels in A549, MCF-7, and Huh-7 cells transfected with pre-mir-control, pre-mir-16, anti-mir-control, and anti-mir-16. **e** Firefly luciferase reporters containing wild-type (WT) or mutant (MUT) miR-16 binding sites in the FEAT 3′-UTR were co-transfected into A549, MCF-7, and Huh-7 cells with pre-mir-control, pre-mir-16, anti-mir-control, and anti-mir-16. Twenty-four hours post-transfection, the cells were assayed using a luciferase assay kit. ***, *P* < 0.001

The correlation between miR-16 and FEAT was further examined by evaluating FEAT expression in human lung adenocarcinoma A549 cells, human breast adenocarcinoma MCF-7 cells, and human hepatocellular carcinoma Huh-7 cells after overexpressing or knocking down miR-16. In these experiments, miR-16 overexpression was achieved by transfecting the cells with pre-mir-16 (a synthetic RNA oligonucleotide duplex mimicking the miR-16 precursor), and miR-16 knockdown was achieved by transfecting the cells with anti-mir-16 (a chemically modified antisense oligonucleotide designed to specifically target mature miR-16). As anticipated, the miR-16 levels were significantly increased in A549, MCF-7, and Huh-7 cells when these cells were transfected with pre-mir-16, whereas the miR-16 levels were decreased when these cells were transfected with anti-mir-16 (Fig. 2a). The expression of the FEAT protein was reduced by the overexpression of miR-16 and increased by the knockdown of miR-16 in A549, MCF-7, and Huh-7 cells (Fig. 2, b and c). To determine the level at which miR-16 influenced FEAT expression, we repeated the above-mentioned experiments and examined the expression of FEAT mRNA after transfection. Although the intracellular level of miR-16 was significantly altered after transfection with pre-mir-16 and anti-mir-16, the alteration of the miR-16 levels did not affect the FEAT mRNA levels (Fig. 2d). These results demonstrate that miR-16 specifically regulates FEAT protein expression at the post-transcriptional level, which is a typical miRNA-mediated regulation mechanism.

To determine whether the negative regulatory effects that miR-16 exerted on FEAT expression were mediated through the binding of miR-16 to the presumed sites in the 3′-UTR of the FEAT mRNA, the full-length FEAT 3′-UTR that contained the sole presumed miR-16 binding site was fused downstream of the firefly luciferase gene in a reporter plasmid. The resulting plasmid was transfected into A549, MCF-7, and Huh-7 cells along with pre-mir-16 or anti-mir-16. As expected, the overexpression of miR-16 resulted in a significant reduction of luciferase reporter activity compared with transfection with pre-scramble control, whereas the inhibition of miR-16 resulted in an increase in reporter activity compared with transfection with anti-scramble control (Fig. 2e). Furthermore, we introduced point mutations into the corresponding complementary sites in the FEAT 3′-UTR to eliminate the predicted miR-16 binding site. This mutated luciferase reporter was unaffected by both the overexpression and knockdown of miR-16 (Fig. 2e). This finding suggests that the binding site strongly contributes to the interaction between miR-16 and FEAT mRNA. In conclusion, our results demonstrate that miR-16 directly recognizes and binds to the 3′-UTR of the FEAT mRNA transcript thereby inhibiting FEAT translation.

### miR-16 promotes the apoptosis of cancer cells by regulating FEAT

**Fig. 3:**
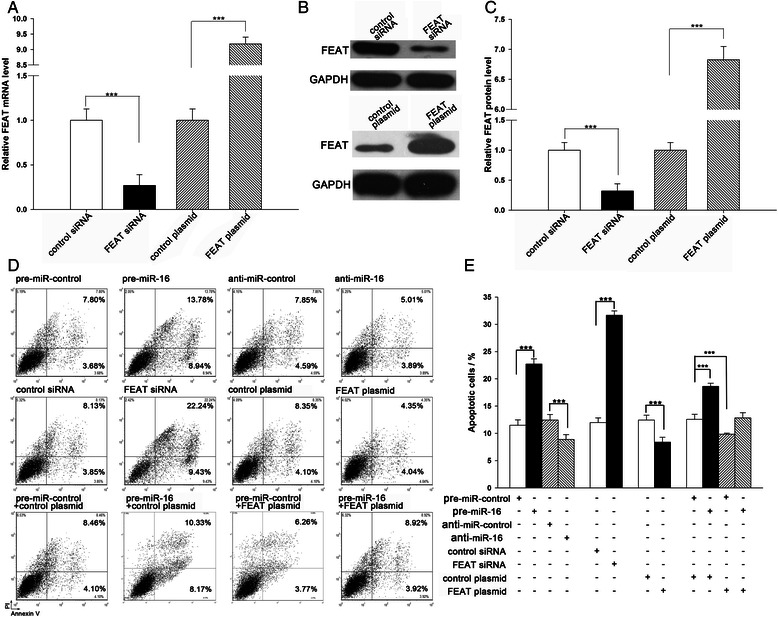
The role of miR-16 targeting FEAT in the regulation of apoptosis of cancer cells. **a** Quantitative RT-PCR analysis of FEAT mRNA levels in A549 cells treated with control siRNA, FEAT siRNA, control plasmid, and FEAT plasmid. **b** and **c** Western blot analysis of FEAT protein levels in A549 cells treated with control siRNA, FEAT siRNA, control plasmid, and FEAT plasmid. B: representative image; C: quantitative analysis. (D and E) A549 cells were transfected with equal doses of pre-mir-control, pre-mir-16, anti-mir-control, anti-mir-16, control siRNA, FEAT siRNA, control plasmid, FEAT plasmid, or with pre-mir-control plus control plasmid, pre-mir-16 plus control plasmid, pre-mir-control plus FEAT plasmid, or pre-mir-16 plus FEAT plasmid. Cell apoptosis profiles were analyzed by flow cytometry. The biparametric histogram shows cells in early (bottom right quadrant) and late apoptotic states (upper right quadrant). Viable cells are double negative (bottom left quadrant). **d**: representative image; **e**: quantitative analysis. ***, *P* < 0.001

We next focused on studying the role of miR-16 in regulating FEAT. Because FEAT is known to be involved in cell apoptosis regulation [3], we investigated whether the overexpression or knockdown of miR-16 or FEAT would impact cell apoptosis in A549 cells using flow cytometry analysis. The efficient overexpression or knockdown of FEAT is shown in Fig. 3, a-c. In support of the notion that FEAT is essential in suppressing apoptosis [3], A549 cells transfected with FEAT siRNA showed a promotion of cell apoptosis (Fig. 3, d and e). In contrast, transfection with the FEAT-overexpressing plasmid, which specially expresses the full-length open reading frame (ORF) of FEAT without the miR-16–responsive 3′-UTR, had an opposite effect on cell apoptosis (Fig. 3, d and e). Subsequently, we assessed the role of miR-16 in cell apoptosis. As expected, A549 cells transfected with pre-mir-16 exhibited a significantly higher rate of cell apoptosis, whereas A549 cells transfected with anti-mir-16 had a lower apoptosis rate (Fig. 3, d and e). Moreover, compared with cells that had been transfected with pre-mir-16, those transfected with pre-mir-16 and the FEAT-overexpressing plasmid exhibited significantly lower apoptosis rates (Fig. 3, d and e), suggesting that miR-16-resistant FEAT is sufficient to rescue the suppression of FEAT by miR-16 and attenuate the pro-apoptotic effect of miR-16 on cancer cells. Taken together, the results indicate that miR-16 can promote cell apoptosis by silencing FEAT.

## Discussion

The significance of overexpressed proteins in cancer is recognized as a potential lead for a variety of diagnostic and therapeutic approaches for cancers. Studies that identify and characterize common oncogenic proteins will hopefully advance molecular-targeted cancer screening and prevention. However, due to the marked heterogeneity and complexity of different types of human cancers, it is rather difficult to identify such proteins that are commonly altered in diverse cancers. Notably, a previously unrecognized protein, FEAT, was recently found to be highly expressed in an unusually wide range of tumors but not expressed in most normal tissues [3], suggesting that FEAT is a ubiquitous protein that is involved in human cancer. FEAT was originally purified from rat livers as a protein that inhibits nuclear apoptosis *in vitro* [3]. *Ex vivo* experiments confirmed that FEAT attenuates apoptotic cell death [3]. Studies have demonstrated that FEAT is highly oncogenic *in vivo* [3]. However, despite these recent advances in our understanding of the important roles of FEAT in cancer progression, the precise molecular mechanism through which FEAT is upregulated during tumorigenesis remains largely unknown. In this study, we showed that silencing FEAT expression using siRNA could promote cell apoptosis in cancer cells, whereas overexpressing FEAT had an opposite effect, validating its role as an essential oncogenic protein during tumorigenesis. Interestingly, we identified discordance between the FEAT protein and mRNA levels in human lung cancer, breast cancer, and hepatocellular cancer tissues. The results suggest that a post-transcriptional regulation mechanism is involved in FEAT repression. One centrally important mode of post-transcriptional regulation is the repression of mRNA transcripts by miRNAs. Therefore, we searched for miRNAs that could target FEAT and experimentally validated miR-16 as a direct regulator of FEAT. The results identified miR-16 as a novel link between the FEAT regulatory pathway and the pathogenesis of cancer. Considering that miR-16 is highly expressed in normal tissues and frequently deleted and downregulated in many types of cancer tissues, the results also explain, at least in part, why FEAT is aberrantly overexpressed in most human cancers but weakly expressed in normal tissues.

In this study, we further investigated whether the cellular phenotypes especially cell apoptosis were regulated by miR-16 targeting FEAT. We showed that miR-16 could suppress FEAT expression and, in turn, promote apoptosis in cancer cells. The results reveal a critical role for miR-16 as a tumor suppressor and pro-apoptotic molecule in carcinogenesis through the repression of FEAT translation. In fact, miR-16 has been reported to act as a tumor-suppressive miRNA in many cancer types [9–16], and multiple apoptosis-related genes are targeted by miR-16, including *BCL-2*, *CCND1*, *CCND3*, and *CCNE1* [9, 13]. We performed KEGG pathway analysis and GO annotation analysis on the experimental validated target genes of miR-16, and the results showed that most of these target genes were indeed anti-apoptotic factors (Additional file 1 Table S1 and S2). An emerging common theme is that multiple targets regulated by a single miRNA can act in concert, rather than individually, to regulate the same biological process, such as apoptosis. The coordinated regulation of many targets by a single miRNA may allow for a prompt cellular response to the apoptosis signals. In this study, it is noted that restoring FEAT expression can successfully attenuate the pro-apoptotic effects of miR-16 on cancer cells, although miR-16 has many other targets. The results suggest that targeting FEAT is a major mechanism by which miR-16 exerts its tumor-suppressive and pro-apoptotic function. Therefore, the modulation of FEAT by miR-16 might explain, at least in part, why the downregulation of miR-16 during carcinogenesis can accelerate cancer progression.

Taken together, this study delineates a novel regulatory network employing miR-16 and FEAT to fine-tune cell apoptosis in lung, breast, and hepatocellular cancer cells. This study may provide a potential novel target for future cancer therapy.

## Additional file

Additional file 1:
**miR-16 promotes the apoptosis of human cancer cells by targeting FEAT.**
