# Supplementary material for: miR-16 promotes the apoptosis of human cancer cells by targeting FEAT
Source: BMC Cancer. 2015 Jun 2;15:448. doi: 10.1186/s12885-015-1458-8 (PMC4450989; doi:10.1186/s12885-015-1458-8)
Supplement: Additional file 1: — miR-16 promotes the apoptosis of human cancer cells by targeting FEAT. [file 12885_2015_1458_MOESM1_ESM.doc]

**Supplementary Materials**

**miR-16 promotes the apoptosis of human cancer cells by targeting FEAT**

**Supplementary Table 1. GO functional annotation of miR-16 target genes.**

| Term | GO Term | Count | % | P-Value | FDR |
| --- | --- | --- | --- | --- | --- |
| GO:0016265 | death | 45 | 7.11 | 1.37E-55 | 2.20E-52 |
| GO:0008219 | cell death | 44 | 6.95 | 4.17E-53 | 6.69E-50 |
| GO:0006915 | apoptosis | 36 | 5.69 | 3.90E-39 | 6.27E-36 |
| GO:0012501 | programmed cell death | 36 | 5.69 | 6.62E-39 | 1.06E-35 |

**Supplement table 2. KEGG pathway enrichment analysis of miR-16 target genes.**

| KEGG ID | KEGG term | Count | Genes | P-value |
| --- | --- | --- | --- | --- |
| hsa05168 | Herpes simplex infection | 5 | TBP，JAK2,IFNG,CYCS,TP53 | 0.002686 |
| hsa05166 | HTLV-I infection | 5 | POLB,TBP,CDKN2A,SLC25A6,TP53 | 0.001327 |
| hsa05164 | Influenza A | 5 | JAK2,IFNG,TNFRSF10A,CYCS,SLC25A6 | 0.002686 |
| hsa05152 | Tuberculosis | 5 | CTSD,HSPD1,JAK2,IFNG,CYCS | 0.00597 |
| hsa05016 | Huntington's disease | 5 | TBP,CYCS,TP53,HTT,SLC25A6 | 0.00597 |
| hsa05203 | Viral carcinogenesis | 4 | POLB,TBP,CDKN2A,TP53 | 0.000871 |
| hsa05200 | Pathways in cancer | 4 | TRAF4,CYCS,CDKN2A,TP53 | 0.000357 |
| hsa05162 | Measles | 4 | JAK2,IFNG,TNFRSF10A,TP53 | 0.001119 |
| hsa05140 | Leishmaniasis | 4 | JAK2,IFNG,HSPD1,CYCS | 0.003812 |
| hsa04115 | p53 signaling pathway | 4 | CYCS，CDKN2A,TP53,STEAP3 | 0.002349 |
| hsa05222 | Small cell lung cancer | 3 | TRAF4,CYCS,TP53 | 0.000301 |
| hsa05169 | Epstein-Barr virus infection | 3 | TBP，IFNG,TP53 | 0.000394 |
| hsa05145 | Toxoplasmosis | 3 | JAK2,IFNG,CYCS | 0.000116 |
| hsa04210 | Apoptosis | 3 | TNFRSF10A,CYCS,TP53 | 0.000152 |
| hsa04151 | PI3K-Akt signaling pathway | 3 | EPHA2,JAK2,TP53 | 0.0002 |
| hsa04142 | Lysosome | 3 | CTSD,SLC11A2,PPT1 | 0.000301 |
| hsa04060 | Cytokine-cytokine receptor interaction | 3 | IFNG，TNFRSF12A，TNFRSF10A | 0.000672 |
| hsa05223 | Non-small cell lung cancer | 2 | CDKN2A,TP53 | 9.51E-05 |
| hsa05220 | Chronic myeloid leukemia | 2 | CDKN2A,TP53 | 3.97E-05 |
| hsa05219 | Bladder cancer | 2 | CDKN2A,TP53 | 1.42E-05 |
| hsa05218 | Melanoma | 2 | CDKN2A,TP53 | 6.15E-05 |
| hsa05214 | Glioma | 2 | CDKN2A,TP53 | 1.22E-05 |
| hsa05212 | Pancreatic cancer | 2 | CDKN2A,TP53 | 1.64E-05 |
| hsa05210 | Colorectal cancer | 2 | CYCS,TP53 | 0.00011 |
| hsa05206 | MicroRNAs in cancer | 2 | CDKN2A,TP53 | 1.42E-05 |
| hsa05161 | Hepatitis B | 2 | CYCS,TP53 | 3.97E-05 |
| hsa05160 | Hepatitis C | 2 | PSME3,TP53 | 0.000127 |
| hsa05014 | Amyotrophic lateral sclerosis (ALS) | 2 | CYCS,TP53 | 0.000196 |
| hsa05012 | Parkinson's disease | 2 | CYCS,SLC25A6 | 6.15E-05 |
| hsa05010 | Alzheimer's disease | 2 | APP,CYCS | 0.000169 |
| hsa04940 | Type I diabetes mellitus | 2 | HSPD1,IFNG | 9.51E-05 |
| hsa04722 | Neurotrophin signaling pathway | 2 | TP53,NGFRAP1 | 0.000127 |
| hsa04650 | Natural killer cell mediated cytotoxicity | 2 | IFNG,TNFRSF10A | 3.43E-05 |
| hsa04630 | Jak-STAT signaling pathway | 2 | JAK2,IFNG | 6.15E-05 |
| hsa04612 | Antigen processing and presentation | 2 | IFNG,PSME3 | 0.000169 |
| hsa04514 | Cell adhesion molecules (CAMs) | 2 | L1CAM,CADM1 | 7.11E-05 |
| hsa04110 | Cell cycle | 2 | CDKN2A，TP53 | 0.000196 |
| hsa04071 | Sphingolipid signaling pathway | 2 | CTSD，TP53 | 1.64E-05 |
| hsa03050 | Proteasome | 2 | IFNG，PSME3 | 3.97E-05 |
| hsa01100 | Metabolic pathways | 2 | PPT1，CYCS | 2.55E-05 |
| hsa05416 | Viral myocarditis | 1 | CYCS | 4.55E-06 |
| hsa05332 | Graft-versus-host disease | 1 | IFNG | 1.29E-06 |
| hsa05330 | Allograft rejection | 1 | IFNG | 1.16E-05 |
| hsa05323 | Rheumatoid arthritis | 1 | IFNG | 2.84E-06 |
| hsa05322 | Systemic lupus erythematosus | 1 | IFNG | 2.53E-05 |
| hsa05321 | Inflammatory bowel disease (IBD) | 1 | IFNG | 2.43E-06 |
| hsa05230 | Central carbon metabolism in cancer | 1 | TP53 | 2.53E-05 |
| hsa05217 | Basal cell carcinoma | 1 | TP53 | 1.10E-06 |
| hsa05216 | Thyroid cancer | 1 | TP53 | 5.32E-06 |
| hsa05215 | Prostate cancer | 1 | TP53 | 4.55E-06 |
| hsa05213 | Endometrial cancer | 1 | TP53 | 2.43E-06 |
| hsa05205 | Proteoglycans in cancer | 1 | TP53 | 6.22E-06 |
| hsa05202 | Transcriptional misregulation in cancer | 1 | TP53 | 8.51E-06 |
| hsa05146 | Amoebiasis | 1 | IFNG | 2.07E-06 |
| hsa05144 | Malaria | 1 | IFNG | 1.16E-05 |
| hsa05143 | African trypanosomiasis | 1 | IFNG | 1.29E-06 |
| hsa05142 | Chagas disease (American trypanosomiasis) | 1 | IFNG | 6.22E-06 |
| hsa05132 | Salmonella infection | 1 | IFNG | 2.53E-05 |
| hsa04978 | Mineral absorption | 1 | SLC11A2 | 2.07E-06 |
| hsa04932 | Non-alcoholic fatty liver disease (NAFLD) | 1 | CYCS | 1.10E-06 |
| hsa04920 | Adipocytokine signaling pathway | 1 | JAK2 | 4.55E-06 |
| hsa04919 | Thyroid hormone signaling pathway | 1 | TP53 | 6.22E-06 |
| hsa04917 | Prolactin signaling pathway | 1 | JAK2 | 2.53E-05 |
| hsa04728 | Dopaminergic synapse | 1 | KIF5A | 1.59E-05 |
| hsa04726 | Serotonergic synapse | 1 | APP | 2.43E-06 |
| hsa04725 | Cholinergic synapse | 1 | JAK2 | 7.28E-06 |
| hsa04660 | T cell receptor signaling pathway | 1 | IFNG | 2.43E-06 |
| hsa04550 | Signaling pathways regulating pluripotency of stem cells | 1 | JAK2 | 1.77E-06 |
| hsa04380 | Osteoclast differentiation | 1 | IFNG | 7.28E-06 |
| hsa04350 | TGF-beta signaling pathway | 1 | IFNG | 1.36E-05 |
| hsa04310 | Wnt signaling pathway | 1 | TP53 | 8.51E-06 |
| hsa04140 | Regulation of autophagy | 1 | IFNG | 8.51E-06 |
| hsa04120 | Ubiquitin mediated proteolysis | 1 | UBE2Z | 1.77E-06 |
| hsa04066 | HIF-1 signaling pathway | 1 | IFNG | 6.22E-06 |
| hsa04062 | Chemokine signaling pathway | 1 | JAK2 | 6.22E-06 |
| hsa04022 | cGMP-PKG signaling pathway | 1 | SLC25A6 | 1.59E-05 |
| hsa04020 | Calcium signaling pathway | 1 | SLC25A6 | 1.77E-06 |
| hsa04015 | Rap1 signaling pathway | 1 | EPHA2 | 7.28E-06 |
| hsa04010 | MAPK signaling pathway | 1 | TP53 | 1.86E-05 |
| hsa03450 | Non-homologous end-joining | 1 | LIG4 | 2.17E-05 |
| hsa03410 | Base excision repair | 1 | POLB | 1.10E-06 |
| hsa03022 | Basal transcription factors | 1 | TBP | 4.55E-06 |
| hsa03018 | RNA degradation | 1 | HSPD1 | 8.51E-06 |
| hsa03010 | Ribosome | 1 | RPS3P3 | 1.10E-06 |
| hsa01212 | Fatty acid metabolism | 1 | PPT1 | 7.28E-06 |
| hsa00970 | Aminoacyl-tRNA biosynthesis | 1 | GARS | 2.17E-05 |
| hsa00920 | Sulfur metabolism | 1 | CYCS | 1.16E-05 |
| hsa00564 | Glycerophospholipid metabolism | 1 | PNPLA6 | 1.29E-06 |
| hsa00062 | Fatty acid elongation | 1 | PPT1 | 1.29E-06 |
